# The TLR4‐IRE1α pathway activation contributes to palmitate‐elicited lipotoxicity in hepatocytes

**DOI:** 10.1111/jcmm.13636

**Published:** 2018-04-19

**Authors:** Chen Shen, Wang Ma, Lei Ding, Songtao Li, Xiaobing Dou, Zhenyuan Song

**Affiliations:** ^1^ Department of Kinesiology and Nutrition University of Illinois at Chicago Chicago IL USA; ^2^ College of Life Science Zhejiang Chinese Medical University Hangzhou China; ^3^ Department of Nutrition and Food Hygiene Public Health College Harbin Medical University Harbin China; ^4^ Department of Pathology University of Illinois Medical Center Chicago IL USA

**Keywords:** ER stress, IRE1α, lipotoxicity, NF‐κB, TLR4

## Abstract

Lipotoxicity induced by saturated fatty acids (SFAs) plays a pathological role in the development of non‐alcoholic fatty liver disease (NAFLD); however, the exact mechanism(s) remain to be clearly elucidated. Toll‐like receptor (TLR) 4 plays a fundamental role in activating the innate immune system. Intriguingly, hepatocytes express TLR4 and machinery for TLR4 signalling pathway. That liver‐specific TLR4 knockout mice are protective against diet‐induced NAFLD suggests that hepatocyte TLR4 signalling pathway plays an important role in NAFLD pathogenesis. Herein, using cultured hepatocytes, we sought to directly examine the role of TLR4 signalling pathway in palmitate‐elicited hepatotoxicity and to elucidate underlying mechanism(s). Our data reveal that palmitate exposure up‐regulates TLR4 expression at both mRNA and protein levels in hepatocytes, which are associated with NF‐κB activation. The inhibition of TLR4 signalling pathway through both pharmacological and genetic approaches abolished palmitate‐induced cell death, suggesting that TLR4 signalling pathway activation contributes to palmitate‐induced hepatotoxicity. Mechanistic investigations demonstrate that inositol‐requiring enzyme 1α (IRE1α), one of three major signal transduction pathways activated during endoplasmic reticulum (ER) stress, is the downstream target of palmitate‐elicited TLR4 activation and mechanistically implicated in TLR4 activation‐triggered cell death in response to palmitate exposure. Collectively, our data identify that the TLR4‐IRE1α pathway activation contributes to palmitate‐elicited lipotoxicity in hepatocytes. Our findings suggest that targeting TLR4‐IRE1α pathway can be a potential therapeutic choice for the treatment of NAFLD as well as other metabolic disorders, with lipotoxicity being the principal pathomechanism.

## 1 INTRODUCTION

Non‐alcoholic fatty liver disease (NAFLD) covers a spectrum of liver diseases ranging from steatosis, non‐alcoholic steatohepatitis (NASH), to final stages of fibrosis/cirrhosis and hepatocellular carcinoma.^1^ With rapidly increased prevalence of NAFLD in the past decades, the demand for effective therapies has never been greater. However, our understanding on the pathogenesis of the disease at the cellular and molecular levels remains speculative, which greatly limited the development of safe and efficacious medicines and therapies.

Lipotoxicity, a term coined to describe the detrimental effects of ectopic lipids accumulation, plays a key role in the pathogenesis of NAFLD.^2^ Although the traditional “two‐hit” hypothesis, proposed eighteen years ago, suggests that hepatic steatosis represents the first hit during the disease development,^3^ emerging evidence supports that elevated circulatory free fatty acid (FFA) concentration, instead of triglyceride accumulation, is the culprit attributing to the disease progression.^4^ Saturated fatty acids (SFA), such as palmitate (16:0) and stearic acid (18:0), induce cell death in a variety of mammalian cell lines, including but not limited to hepatocytes, macrophages and vascular smooth muscle cells (VSMCs).^5^, ^6^, ^7^, ^8^, ^9^ Palmitate is the most common FFAs found in animals, plants and microorganisms and one of the most abundant FFAs in human circulation.^10^ The mechanisms underlying palmitate‐induced lipotoxicity remain obscure. Accumulated evidence supports that the metabolites of palmitate, for example, ceramides and phosphatidic acid, contribute to its lipotoxic effect, which have been reported in pancreas and cardiac vascular tissues.^11^, ^12^ In hepatocytes, in vitro studies suggest that multiple pathways are altered upon palmitate exposure, including ceramide synthesis, JNK activation, oxidative stress and endoplasmic reticulum (ER) stress, with results varying, or even conflicting.^13^, ^14^, ^15^, ^16^, ^17^, ^18^

The ER is an organelle playing a critical role in cellular protein folding and assembly. Accumulation of unfolded or misfolded proteins in the ER lumen during certain pathophysiological conditions leads to the activation of a group of signal transduction pathways, which is collectively named unfolded protein response (UPR) or ER stress.^19^ Among three major signal transduction pathways activated during UPR, including PKR‐like ER kinase (PREK), inositol‐requiring enzyme 1α (IRE1α) and activating transcription factor 6 (ATF6), IRE1α is the most conserved one, acting as a protein kinase and endoribonuclease.^20^ In response to ER stress inducers, IRE1α undergoes homodimerization and autophosphorylation to achieve its endoribonuclease (RNase) activity, leading to an unconventional splicing of the mRNA encoding X‐box binding protein 1 (XBP‐1) by removing a 26‐base intron,^20^, ^21^ an indicative of IRE1α activation.

Other than directly inducing cell death, SFAs, specifically palmitate, also play a pivotal role in the induction of inflammatory response via acting as a Toll‐like‐receptor (TLR) 4 ligand, whereby contributing to the development of whole body insulin resistance.^22^ Palmitate‐elicited TLR4 signalling pathway activation has been reported in both adipocytes and macrophages and TLR4 deletion blunted palmitate‐induced inflammatory signalling in these cells,^22^ suggesting that TLR4 signalling pathway plays a pivotal role in palmitate‐elicited inflammatory response. The involvement of TLR4 activation in palmitate‐induced cell death was recently reported in macrophages.^22^ TLR4 activation under lipotoxic conditions led to macrophage cell death, which was mediated by TRIF, one of the downstream targets of TLR4 signalling.^23^ Several other studies using whole body TLR4 knockout mice consistently demonstrated that TLR4 pathway was critically involved in NASH progression in various animal models.^24^, ^25^ Although the majority of the work relating TLR4 activation to NAFLD progression focused on macrophages, a very recent in vivo study using tissue‐specific TLR4 knockout mice revealed that hepatocyte‐specific TLR4 knockout mice exhibited improved glucose tolerance, enhanced insulin sensitivity and ameliorated hepatic steatosis after a high‐fat diet challenge.^26^ Thus, TLR4 activation in hepatocytes and its significance in the process of lipotoxicity and the pathogenesis of NAFLD require further evaluation. Interestingly, several recent studies revealed that, in monocytes and macrophages, TLR4 activation was associated with the activation of IRE1α, one of the three branches of downstream UPR/ER stress signalling.^27^ However, whether this is also the scenario for hepatocytes and importantly whether this pathway contributes to palmitate‐induced lipotoxicity in hepatocytes remain unknown.

In this study, we conducted in vitro experiments to study the role of TLR4 activation in palmitate‐induced lipotoxicity and sought to elucidate underlying mechanisms behind it. Our study reveals that TLR4 signalling pathway contributes to palmitate‐induced cell death in hepatocytes via activating IRE1α endoribonuclease activity.

## 2 MATERIALS AND METHODS

### 2.1 Chemicals

Chemicals including palmitate (P0500), bovine serum albumin (BSA, A7030), myriocin (M1177), bay11‐7082 (B5556), STF‐083010 (SML0409) and DMSO (D2650) were purchased from Sigma‐Aldrich. Other chemicals used in this study were purchased as follows: CLI‐095 (InvivoGen, 14I24‐MM) and TLR4 neutralizing antibody (PAb hTLR4, InvivoGen, pab‐hstlr4). Palmitate‐BSA conjugates were prepared step by step as follows: powdered palmitate was dissolved in ethanol and saponified with sodium hydroxide. The sodium salt was dried, resuspended in saline and heated at 80°C until completely dissolved. While the solution was still warm, isovolumetric 20% (w/v) BSA was added and the mixture was stirred at 50°C for 4 hours to allow palmitate to bind to BSA. The palmitate‐BSA complex (3 mmol/L fatty acid: 1.5 mmol/L BSA; molar ratio, 2:1) was then sterilized by filtering using 0.2‐mm‐diameter filter and aliquoted for future use. In all the experiments except specific explanations, the control group was exposed to an equal amount of solvent (eg BSA, ethanol, DMSO).

### 2.2 Cell culture

HepG2 (HB‐8065) cells were obtained from the American Type Culture Collection (Manassas, VA). Primary mouse hepatocytes were purchased from Thermo Fisher Scientific (MSFN24). Cells were cultured using Dulbecco's modified Eagle medium (DMEM from Sigma‐Aldrich, D5648) containing 10% (v/v) foetal bovine serum (PAA Laboratories, A15‐701), 2 mmol/L glutamine (Sigma‐Aldrich, G3126), 100 U/mL penicillin and 100 μg/mL streptomycin (Life technologies, 15140‐122) at 37°C in a humidified O_2_/CO_2_ (95:5) atmosphere. All experiments were repeated at least 3 times.

### 2.3 RNA interference

Cultured HepG2 cells were transfected with human TLR4 siRNA (Thermo Fisher Scientific, AM16708) or human IRE1alpha siRNA (Santa Cruz Biotechnology, sc‐40705), using Lipofectamine 2000 according to the manufacturer's instructions. In the control group, cells were transfected with scrambled siRNA (Santa Cruz Biotechnology, sc‐37007).

### 2.4 LDH release assay

2 × 10^5^/mL cells were seeded in 24‐well plates and cultured overnight. After indicated treatments, culture medium was collected and detected using an LDH assay kit (Thermo Scientific Inc., NC9674653) according to the manufacturer's instructions. The absorption at OD510 was measured using a SPECTRAmax 340PC microplate reader. The relative LDH release levels were scaled as folds of the controlled untreated group.

### 2.5 MTT assay

Cells were seeded in 96‐well plates at a density of 2 × 10^4^/well and cultured overnight. After indicated treatments, 25 μL of fresh MTT (3‐(4, 5‐dimethylthiazol‐2‐yl)‐2, 5‐diphenyltetrazolium bromide, 5 mg/mL) was added into each well. The cells were incubated at 37°C for 2 hours to allow incorporation and conversion of MTT to the formazan derivative. The formazan derivative was solubilized by the addition of 100 μL of lysis buffer (20% SDS in 50% dimethylformamide, pH 4.7). After incubation for 24 hours at 37°C, the absorbance values were measured at 570 nm using a SPECTRAmax 340PC microplate reader.

### 2.6 Total RNA extraction and quantitative real‐time PCR

2 × 10^5^ cells/mL were seeded in 24‐well plates and cultured overnight. After the indicated treatments, 1.0 mL of TRIzol (Invitrogen, Carlsbad, CA) was added into each well and transferred into 1.5‐mL tubes. Total RNA was isolated according to the manufacturer's protocol with an additional phenol/chloroform extraction. Isolated RNA was resuspended in nuclease‐free water and quantified by a Thermo Scientific NanoDrop 2000 spectrophotometer. For each sample, 1.0 μg of total RNA was reverse transcribed using a high‐capacity complementary DNA reverse transcription kit as described by the manufacturer (Applied Biosystems, Foster City, CA). RT‐PCR products were stored at −80°C.

Complimentary DNA obtained from RT‐PCR was used as template in a 25 μL polymerase chain reaction (PCR) solution system containing 10.5 μL dH_2_O, 1 μL 10 μmol/L gene‐specific primers and 12.5 μL 1 × Power SYBR Green PCR master mix (4367659, Applied Biosystems, Foster City, CA). Polymerase chain reaction amplification was conducted in Bio‐Rad Hard‐Shell 96‐well PCR plates (Bio‐Rad, Cat#HSS9601) on an Agilent Technologies Stratagene Mx3005P under the following conditions: initial denaturation and enzyme activation for 10 minutes at 95°C, followed by 45 cycles of 95°C for 15 seconds, 58°C for 30 seconds, 72°C for 30 seconds and then 95°C for another 10 seconds. Data were analysed using ∆∆Ct method, with Ct stands for threshold cycle. Rox in the master mix was used as reference dye. The copy number of each unknown sample for each gene was standardized to a housekeeping gene (human 18S ribosomal RNA) to control for differences in loading and quality. The relative expression levels were scaled such that the expression level of the time‐matched control group was equal to 1.

The following primers were customized and synthesized by Thermo Fisher Scientific and used in quantitative real‐time PCR:

human 18S forward, GGC CCT GTA ATT GGA ATG AGT C;

human 18S reverse, CCA AGA TCC AAC TAC GAG CTT;

human XBPu forward, CCT GGT TGC TGA AGA GGA GG;

human XBPu reverse, CCA TGG GGA GAT GTT CTG GAG;

human XBPs forward, AAA CAG AGT AGC AGC TCA GAC TG;

human XBPs reverse, TCC TTC TGG GTA GAC CTC TGG GAG;

human CIAP2 forward, TGG AAG CTA CCT CTC AGC CTA C;

human CIAP2 reverse, GGA ACT TCT CAT CAA GGC AGA.

The following primers were commercially available ones and purchased from Qiagen: human TLR4 (NM_003266), Bcl‐2 (NM_000633), TNF‐alpha (NM_000594) and IP‐10 (NM_0001565).

### 2.7 NF‐κB p65 binding activity assay

Nuclear lysate was used following the manufacturer's instruction for NF‐κB p65 Transcription Factor Assay Kit (ab133112, Abcam) for determination of nuclear p65 binding activity.

### 2.8 Statistical analysis

All data were expressed as mean ± SD. Statistical analysis was performed with a one‐way ANOVA and was analysed further by post hoc test with Fisher's least significant difference (LSD) for statistical differences. Differences between treatments were considered to be statistically significant at *P* < .05.

## 3 RESULTS

### 3.1 Palmitate exposure up‐regulates TLR4 expression and activates TLR4 signalling pathway

**Figure 1:**
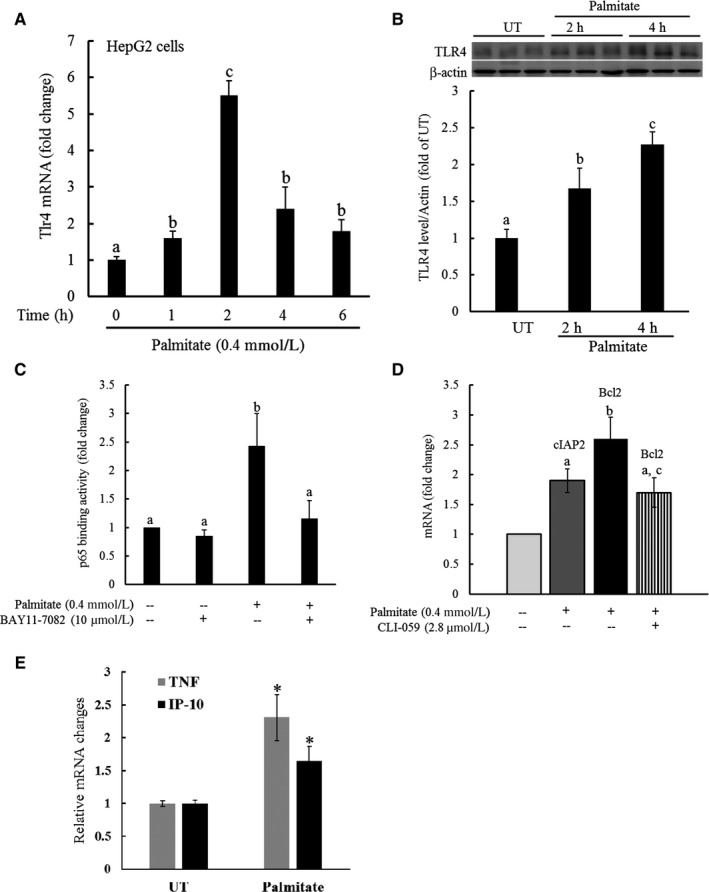
Palmitate exposure up‐regulates TLR4 expression and activates TLR4 signalling pathway. A, HepG2 cells were treated with palmitate at 0.4 mM for various durations. TLR4 gene expression was quantified by real‐time RT‐PCR. All values are denoted as means ± SD from three or more independent experiments. Bars with different characters differ significantly (*P* < .05). B, HepG2 cells were treated with palmitate at 0.4 mmol/L for either 2 or 6 hours. TLR4 protein abundance was quantified by Western blot. C, HepG2 cells were treated with palmitate at 0.4 mM with/without 1 hour pre‐treatment with BAY11‐7082, a NF‐κB inhibitor, for 2 hours. Nuclear proteins were isolated and subjected to ELISA for the determination of p65‐DNA‐binding activity. All values are denoted as means ± SD from three or more independent experiments. Bars with different characters differ significantly (*P* < .05). D, HepG2 cells were treated with palmitate at 0.4 mmol/L at the presence/absence of CLI‐059, a TLR4 inhibitor, for 16 hours. Gene expression of cIAP2 and Bcl2, two NF‐κB target proteins, was quantified by real‐time RT‐PCR. All values are denoted as means ± SD from three or more independent experiments. Bars with different characters differ significantly (*P* < .05). E, HepG2 cells were treated with palmitate at 0.4 mmol/L for 16 hours. Gene expressions of TNF‐alpha and IP‐10 were quantified by real‐time RT‐PCR. All values are denoted as means ± SD from three or more independent experiments. *P < .05 vs untreated cells

Hepatocytes express TLR4 and hepatocyte‐specific TLR4 knockout mice on a high‐fat/high‐fructose diet develop less severe NAFLD,^22^, ^26^ suggesting that TLR4 signalling pathway in hepatocytes plays an important role in NAFLD pathogenesis. Herein, we directly examined the effect of palmitate exposure on TLR4 expression and downstream signalling pathway activation in hepatocytes. HepG2 cells were treated with 0.4 mmol/L palmitate for indicated exposure durations. TLR4 gene expression and protein abundance were quantified by real‐time RT‐PCR and Western blot, respectively. As shown in Figure 1A, TLR4 mRNA was significantly increased in response to palmitate challenge, which peaked at 2‐hour time‐point (~4‐fold increase over untreated cells). The elevation of TLR4 gene expression was retained during the whole 6‐hour experimental period. In accordance, increased protein abundance of TLR4 was observed after 2‐hour palmitate exposure (Figure 1B)

**Figure 2:**
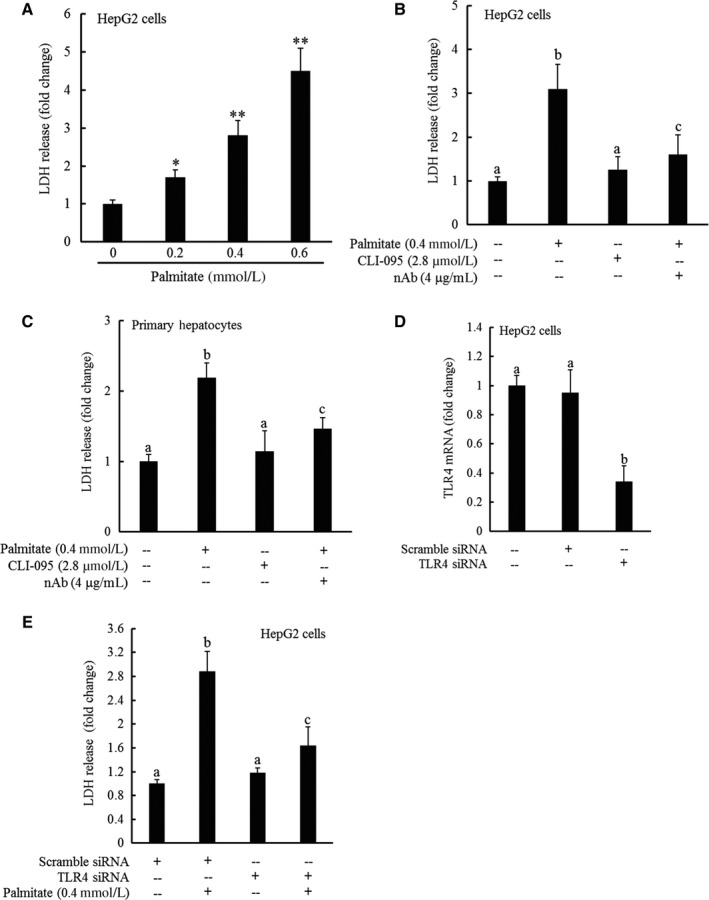
TLR4 signalling pathway activation contributes to palmitate‐induced cell death. HepG2 cells were treated with palmitate at 0, 0.2, 0.4 and 0.6 mmol/L for overnight. Cell viability was determined by LDH release measurement (A). All values are denoted as means ± SD from three or more independent experiments. *P < .05 vs untreated cells; **P < .01 vs untreated cells. B, HepG2 cells were treated with 0.4 mmol/L palmitate at the presence/absence of CLI‐059, a TLR4 inhibitor or TLR4 neutralizing antibody for overnight. Cell viability was determined by LDH release measurement. All values are denoted as means ± SD from three or more independent experiments. Bars with different characters differ significantly (*P* < .05). C, Primary mouse hepatocytes were treated with 0.4 mmol/L palmitate at the presence/absence of CLI‐059, a TLR4 inhibitor or TLR4 neutralizing antibody for overnight. Cell viability was determined by LDH release measurement. All values are denoted as means ± SD from three or more independent experiments. Bars with different characters differ significantly (*P* < .05). D‐E, HepG2 cells were transfected with either scramble siRNA or siRNA for TLR4 for overnight, followed by 0.4 mmol/L palmitate exposure. TLR4 gene expressions were determined by real‐time PCR (D), and cell viability was measured by LDH release measurement 16 hours later. All values are denoted as means ± SD from three or more independent experiments. Bars with different characters differ significantly (*P* < .05)

TLR4 signalling cascade includes both Myd88‐dependent and Myd88‐independent pathways, with NF‐κB activation being a major downstream event of TLR4 signalling pathway activation. To determine whether TLR4 up‐regulation in response to palmitate exposure is concomitant with the activation of its signalling pathway, we next examined the effect of palmitate exposure on NF‐κB p65‐DNA‐binding activity by ELISA using nuclear proteins. HepG2 cells were treated with or without Bay 11‐7082 (10 μmol/L, an NF‐κB inhibitor) for 1 hour, followed by a 2‐hour 0.4 mmol/L palmitate exposure. Nuclear proteins were extracted and subjected to ELISA for the determination of p65‐DNA‐binding activity. As shown in Figure 1C, palmitate exposure resulted in a more than twofold increase in p65‐DNA‐binding activity, which was abrogated by Bay 11‐7082 pre‐treatment. Furthermore, we showed that palmitate exposure significantly increased gene expression of cIAP2 and Bcl‐2, two NF‐κB target proteins (Figure 1D). Importantly, palmitate‐induced up‐regulation of NF‐κB target genes was blunted by CLI‐059 (1 μg/mL), a specific TLR4 inhibitor (Figure 2D), suggesting that palmitate activates TLR4 signalling pathway in hepatocytes. Finally, the expressions of two signature genes in response to TLR4 signalling pathway activation, TNF‐α (Myd88‐dependent pathway) and IP‐10 (Myd88‐independent pathway), were examined. As shown in Figure 2E, both genes were up‐regulated by palmitate exposure.

### 3.2 TLR4 signalling pathway activation contributes to palmitate‐induced cell death

We previously reported that palmitate exposure induced cell death in HepG2 cells.^28^, ^29^ In this study, both HepG2 cells, a human hepatoma cell line and primary mouse hepatocytes were employed to generalize our key observations in hepatocytes. Consistent with our previous reports, a dose‐dependent decrease in cell viability was observed in HepG2 cells exposed to palmitate (Figure 2A). To determine whether TLR4 signalling pathway activation contributes to the palmitate‐induced cell death, we blockaded TLR4 signalling by three approaches, including pharmacological inhibitor (CLI‐095) pre‐treatment, neutralizing antibody pre‐exposure and overnight transfection with TLR4 siRNA. As shown in Figure 2B and C, both CLI‐095 and neutralizing antibody pre‐treatment attenuated palmitate‐induced cell death in hepatocytes. To exclude the possible non‐specific effects caused by chemical inhibitor and neutralizing antibody, HepG2 cells were transfected with either control (scramble) siRNA or siRNA for TLR4 for overnight, followed by palmitate exposure at 0.4 mmol/L for 16 hours. Similar to that observed with chemical blockers, TLR4 knockdown (Figure 2D) protected HepG2 cells against palmitate‐induced cell death (Figure 2E).

### 3.3 Palmitate‐induced cell death is independent of NF‐κB activation

**Figure 3:**
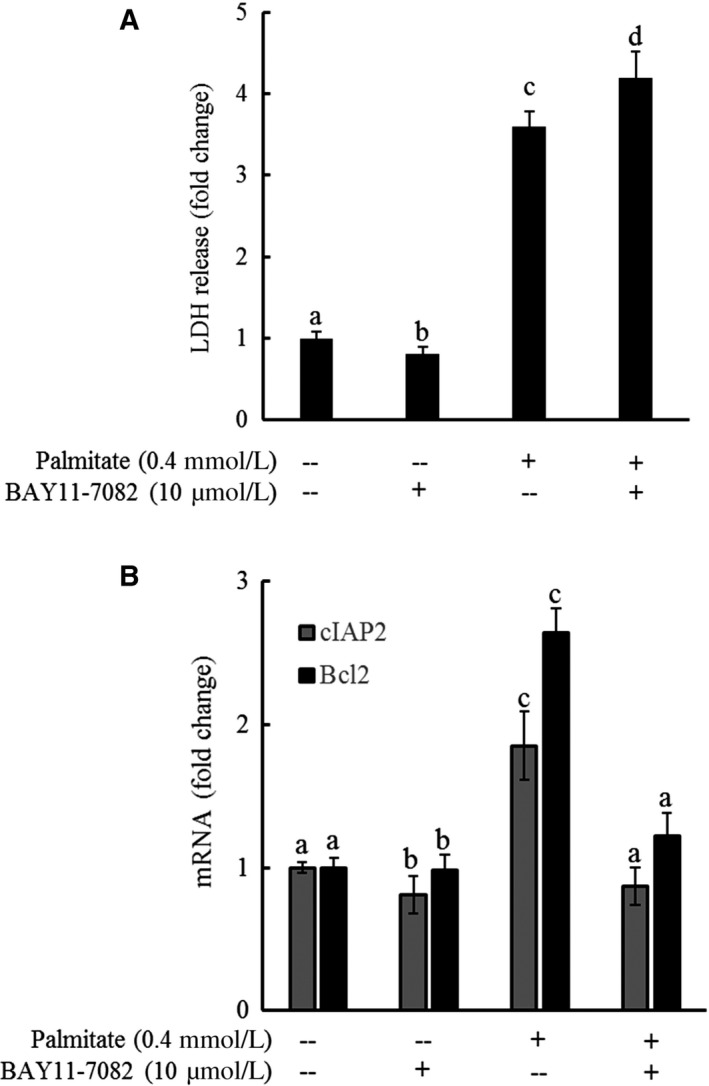
Palmitate‐induced cell death is independent of NF‐κB activation. HepG2 cells were incubated with Bay 11‐7082, a well‐established NF‐κB inhibitor, for 1 hour prior to palmitate (0.4 mmol/L) exposure for 16 hours. A, LDH release. B, Gene expression of NF‐κB target genes. All values are denoted as means ± SD from three or more independent experiments. Bars with different characters differ significantly (*P* < .05)

Given the fact that NF‐κB activation is a main downstream event in response to TLR4 ligand stimulation, we reasoned that NF‐κB might be mechanistically involved in palmitate‐induced lipotoxicity. To test our hypothesis, HepG2 cells were incubated with Bay 11‐7082, a well‐established NF‐κB inhibitor, for 1 hour prior to palmitate exposure, followed by cell death assessment by measuring LDH release after 16 hours. As shown in Figure 3A, Bay 11‐7082 pre‐treatment conferred no protection against palmitate‐induced cell death while NF‐κB activation was profoundly suppressed, evidenced by the significant down‐regulation of target genes (Figure 3B).

### 3.4 IRE1α activation contributes to palmitate‐induced cell death

**Figure 4:**
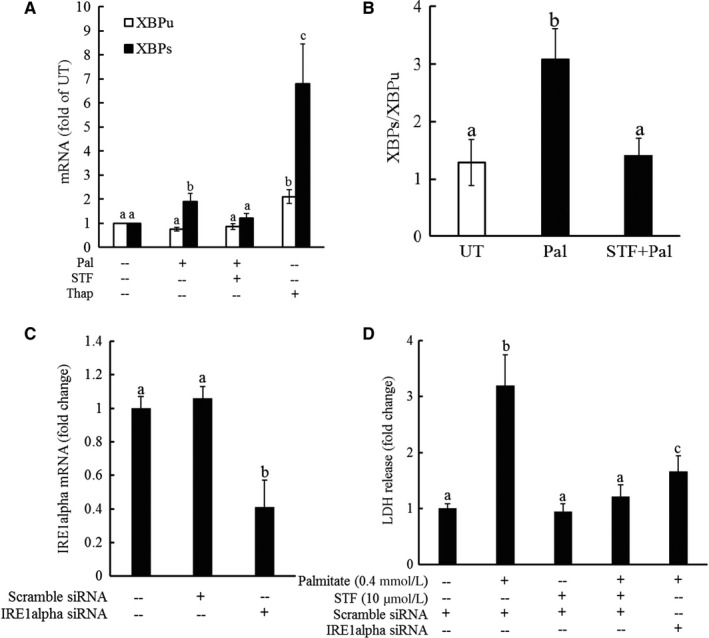
IRE‐1α activation contributes to palmitate‐induced cell death. A, HepG2 cells were exposed to 0.4 mmol/L palmitate at the presence/absence of STF083010, a specific inhibitor of IRE1α's endonuclease activity, for one hour. Total RNA was extracted 8 hours later for the measurement of both unspliced XBP1 (XBP1u) and spliced XBP1 (XBP1s) mRNA by real‐time RT‐PCR. Cell death was determined after 16‐hour palmitate exposure by LDH release assay. Thapsigargin, a well‐established potent ER stress inducer, was used as a positive control for the specificity of the primer sequence of both unspliced XBP1 (XBP1u) and spliced XBP1 (XBP1s). All values are denoted as means ± SD from three or more independent experiments. Bars with different characters differ significantly (*P* < .05). B, XBP1s to XBP1u ratio. Bars with different characters differ significantly (*P* < .05). C‐D, HepG2 cells were transfected with either scramble siRNA or siRNA for IRE1α for overnight, followed by 0.4 mmol/L palmitate exposure. IRE1α gene expressions were determined by real‐time PCR (C), and cell viability was measured by LDH release measurement 16 hours later. All values are denoted as means ± SD from three or more independent experiments. Bars with different characters differ significantly (*P* < .05)

IRE1α is an ER transmembrane protein with both kinase and endoribonuclease activity.^20^ Upon ER stress, its activation leads to an unconventional cleavage of the XBP1 mRNA by removal of an intron which results in the change in the open reading frame, forming spliced XBP1 (XBP1s), which encodes a transcription activator that drives the transcription of genes participate in ER protein folding.^20^, ^21^ To examine whether IRE1α activation is implicated in palmitate cytotoxicity, HepG2 cells were incubated with STF083010 (a specific inhibitor of IRE1α's endonuclease activity) to block endogenous XBP1 cleavage for one hour prior to palmitate exposure. Total RNA was extracted for real‐time RT‐PCR after an 8‐hour treatment, and cell death was determined after 16 hours. Thapsigargin, a well‐established potent ER stress inducer, was used as a positive control for the specificity of the primer sequence of both unspliced XBP1 (XBP1u) and spliced XBP1 (XBP1s). Figure 4A shows the gene expression of both XBP1u and XBP1s determined by real‐time PCR. Thapsigargin robustly induced the splicing of XBP1, confirming the activation of IRE1α pathway in response to ER stress. Palmitate exposure induced a significant elevation of XBP1s, while XBP1u expression was not significantly affected, leading to a profound increase in XBP1s to XBP1u ratio (Figure 4B), the indicative of IRE1α activation. Palmitate‐triggered XBP1 splicing was almost completely blocked by STF083010 pre‐treatment (Figure 4A and B), further confirming that IRE1α was activated by palmitate exposure.

The implication of IRE1α activation in palmitate‐induced cell death was subsequently investigated via suppressing IRE1α pathway both pharmacologically and genetically. HepG2 cells were incubated with STF083010 for 1 hour or transfected with IRE1α siRNA for overnight prior to 0.4 mmol/L palmitate exposure. Cell death was determined by LDH release measurement after 16 hours. As shown in Figure 4C and D, both pharmacological suppression of IRE1α activity and genetic knockdown of IRE1α expression via siRNA transfection abolished palmitate‐induced cell death.

### 3.5 TLR4 pathway activation contributes to palmitate‐triggered IRE1α activation

**Figure 5:**
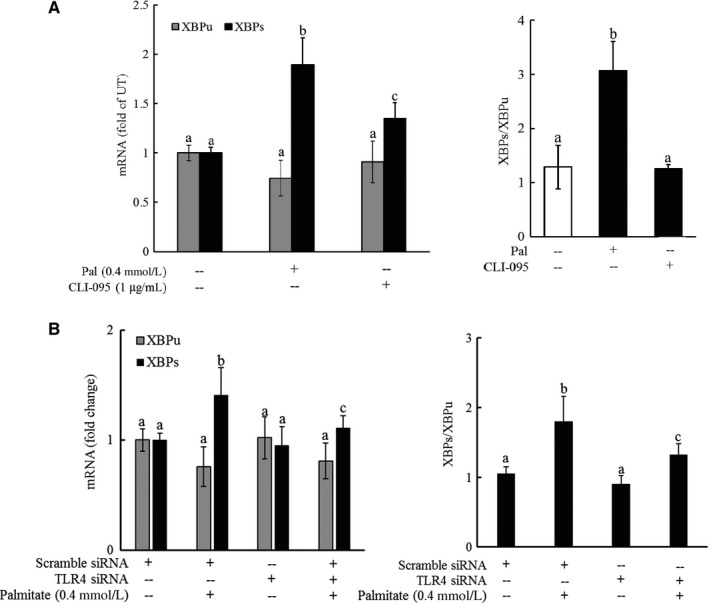
TLR4 pathway activation contributes to palmitate‐triggered IRE1α activation. A, HepG2 cells were pre‐treated with or without CLI‐095, a chemical inhibitor of TLR4, for 1 hour, followed by palmitate exposure. Total RNA was extracted after 6 hours, and mRNA levels of XBP1u and XBP1s were determined by real‐time RT‐PCR and XBP1s/XBP1u calculated. All values are denoted as means ± SD from three or more independent experiments. Bars with different characters differ significantly (*P* < .05). B, HepG2 cells were transfected with either scramble siRNA or TLR4 siRNA for overnight, followed by palmitate exposure. Total RNA was extracted after 6 hours, and mRNA levels of XBP1u and XBP1s were determined by real‐time RT‐PCR and XBP1s/XBP1u calculated. All values are denoted as means ± SD from three or more independent experiments. Bars with different characters differ significantly (*P* < .05)

As both TLR4 signalling pathway blockage and IRE1α suppression conferred protection against palmitate‐induced cell death in hepatocytes, we posited that TLR4 pathway activation might be mechanistically involved in palmitate‐triggered IRE1α activation. To test our hypothesis, HepG2 cells were pre‐treated with CLI‐095 (TLR4 inhibitor) for 1 hour or transfected with TLR4 siRNA for overnight, followed by palmitate exposure. Total RNA was extracted after 6 hours, and the gene expression was determined by real‐time RT‐PCR. As shown in Figure 5A and B, palmitate exposure activated IRE1α, which was evidenced by a markedly increase in XBP1s mRNA level, leading to an approximate 2.5‐fold increase in the XBP1s to XBP1u ratio (Figure 5A and B). Interestingly, both TLR4 inhibitor and TLR4 siRNA blunted palmitate‐elicited XBP1 splicing (Figure 5A and B), suggesting that TLR4 signalling pathway activation is mechanistically involved in palmitate‐induced IRE1α activation.

## 4 DISCUSSION

In the present study, we provide evidence supporting that the TLR4‐IRE1α pathway activation is critically involved in palmitate‐induced lipotoxicity in hepatocytes. We demonstrated that palmitate exposure up‐regulated TLR4 expression and activated its downstream signalling pathway. Importantly, we found that TLR4 inhibition protected hepatocytes against lipotoxicity provoked by palmitate exposure. Our study further revealed that palmitate exposure of hepatocytes led to TLR4‐dependent activation of IRE1α and IRE1α inhibition prevented hepatocytes from palmitate‐elicited hepatotoxicity.

Hepatocyte cell death induced by SFAs, namely lipotoxicity, plays a pathological role in the development of NAFLD; however, the underlying mechanism(s) remains to be fully elucidated and are believed to be multifactorial. The recent report that hepatocyte‐specific TLR4 knockout mice were protected against long‐term high‐fat diet‐induced NAFLD^26^ prompted us to explore the role of TLR4 signalling in palmitate‐induced hepatotoxicity. TLR4, a key receptor in the regulation of inflammation, is activated upon binding with its ligands, for example, the bacterial lipopolysaccharide (LPS). Once binding with its ligand, TLR4 undergoes oligomerization and recruits its downstream adaptors through interactions with the Toll‐interleukin‐1 receptor (TIR) domains to activate either the myeloid differentiation primary response protein 88 (MyD88)‐dependent or MyD88‐independent pathway.^22^ The MyD88‐dependent pathway involves an early phase of NF‐κB activation, leading to the production of inflammatory cytokines, while the MyD88‐independent pathway involves the later phase that activates the interferon regulatory factor (IRF3).^30^ In monocytes, palmitate elicited the production of pro‐inflammatory cytokines via activation TLR4 pathway, linking innate immunity and fatty acid‐induced insulin resistance.^31^, ^32^ Hepatocytes express TLR4 and complete machinery for TLR4 signalling transduction pathway^33^; however, the role of TLR4 pathway activation in fatty acid‐induced hepatotoxicity remains to be characterized. The data obtained in the present study clearly showed that, in hepatocytes, palmitate exposure resulted in a more than fivefold increase in TLR4 expression as early as 2 hours post‐exposure when compared with the untreated cells. This was concomitant with the subsequent activation of NF‐κB, the major downstream target of TLR4 signalling pathway, suggesting that palmitate activates TLR4 signalling pathway in hepatocytes, similar to that previously observed in immune cells.^23^ Intriguingly, TLR4 inhibition via either pharmacological or genetic approaches rescued hepatocytes from palmitate‐induced lipotoxicity. Our data altogether highlighted that TLR4 signalling pathway activation is mechanistically involved in palmitate‐induced lipotoxicity in hepatocytes.

The IRE1α is the only identified ER stress sensor conserved in yeast, plants and animals^20^; however, in addition to being activated by the canonical UPR/ER stress pathway, accumulating evidence suggests the existence of the alternative mechanisms for IRE1α activation. Indeed, in macrophages, both TLR4 and TLR2 were able to specifically activate IRE1α to promote pro‐inflammatory cytokine expression in spite of the absence of an ER stress response.^34^, ^35^ In the liver, fructose promotes hepatic de novo lipogenesis via eliciting ER stress‐independent IRE1α‐XBP1 activation.^36^ Previous studies, including ours, reported that palmitate exposure instigated UPR/ER stress, which was attributed to the cell death in hepatocytes.^28^ In this study, we demonstrated that IRE1α inhibition almost completely prevented palmitate‐induced cell death, suggesting that IRE1α pathway activation contributes to lipotoxicity in hepatocytes. Our observations that both TLR4 and IRE1α inhibition conferred protection against palmitate‐induced cell death emboldened us to investigate the potential existence of the link between TLR4 and IRE1α in hepatocytes in response to palmitate challenge. We showed that, under the circumstance of TLR4 inhibition, palmitate‐induced IRE1α activation was significantly compromised. These results provided initial evidence supporting the notion that TLR4 pathway is mechanistically involved in palmitate‐triggered IRE1α activation, whereby leading to palmitate‐induced lipotoxicity in hepatocytes.

NF‐κB activation is a major downstream event in response to TLR4 activation. It is thus conceivable to posit that NF‐κB activation may contribute to palmitate‐induced cell death in hepatocytes. However, our observation that NF‐κB inhibitor pre‐treatment failed to protect hepatocytes against palmitate‐induced cell death suggested that an NF‐κB‐independent pathway underlies the role of TLR4 activation in palmitate‐induced cell death. This observation is indeed in line with a previous report which demonstrated that TLR4 signalling mediated palmitate‐instigated up‐regulation of gluconeogenic gene expression in hepatocytes in a NF‐κB‐independent mechanism^37^. In that study, Mamedova et al. elegantly showed that neither the TLR4 decoy peptide nor siRNA silencing of TLR4 affected palmitate‐induced NF‐κB activation. Further investigation is warranted to elucidate fundamental mechanism(s) linking sequential TLR4 and IRE1α activations in response to palmitate challenge.

In summary, this study provides direct experimental evidence that sequential TLR4‐IRE1α signalling pathway contributes palmitate‐induced cell death in hepatocytes. Targeting this pathway may represent a potential therapeutic choice for the treatment of liver diseases with lipotoxicity being an underlying pathological mechanism, including both NAFLD and alcoholic liver disease.

## CONFLICT OF INTEREST

None.
